# Integration of a Systems Biological Network Analysis and QTL Results for Biomass Heterosis in *Arabidopsis thaliana*

**DOI:** 10.1371/journal.pone.0049951

**Published:** 2012-11-16

**Authors:** Sandra Andorf, Rhonda C. Meyer, Joachim Selbig, Thomas Altmann, Dirk Repsilber

**Affiliations:** 1 Department Genetics and Biometry, Bioinformatics and Biomathematics Group, Leibniz Institute for Farm Animal Biology (FBN), Dummerstorf, Germany; 2 Department of Medicine, Institute for Biostatistics and Informatics in Medicine and Ageing Research, University of Rostock, Rostock, Germany; 3 Department of Molecular Genetics, Leibniz Institute of Plant Genetics and Crop Plant Research (IPK), Gatersleben, Germany; 4 Bioinformatics Chair, Institute for Biochemistry and Biology, University of Potsdam, Potsdam, Germany; John Innes Centre, United Kingdom

## Abstract

To contribute to a further insight into heterosis we applied an integrative analysis to a systems biological network approach and a quantitative genetics analysis towards biomass heterosis in early *Arabidopsis thaliana* development. The study was performed on the parental accessions C24 and Col-0 and the reciprocal crosses. In an over-representation analysis it was tested if the overlap between the resulting gene lists of the two approaches is significantly larger than expected by chance. Top ranked genes in the results list of the systems biological analysis were significantly over-represented in the heterotic QTL candidate regions for either hybrid as well as regarding mid-parent and best-parent heterosis. This suggests that not only a few but rather several genes that influence biomass heterosis are located within each heterotic QTL region. Furthermore, the overlapping resulting genes of the two integrated approaches were particularly enriched in biomass related pathways. A chromosome-wise over-representation analysis gave rise to the hypothesis that chromosomes number 2 and 4 probably carry a majority of the genes involved in biomass heterosis in the early development of *Arabidopsis thaliana*.

## Introduction

The heterosis phenomenon, also known as hybrid vigor, was discovered in the early 20th century [1]. It describes the superiority in fitness-related traits of F1 hybrids compared to their parental homozygous lines [2]. Mid-parent heterosis (MPH) is the difference between the trait value of the hybrid and the average trait value of the two parental inbred lines, while best-parent heterosis (BPH) is the difference between the hybrid and the better of the homozygous parents. Even though the plant breeding interest in heterosis is high, the underlying genetic and molecular mechanisms are still not fully understood.

In this work, we try to further approach the molecular basis of heterosis by integrating the results of our previously proposed systems biological hypothesis towards the understanding of heterosis on the molecular level [3], [4] with the outcome of a quantitative genetics study for biomass heterosis in early development of *Arabidopsis thaliana* by Meyer *et al.* [5]. In both analyses the same two parental accessions, C24 and Columbia (Col-0), which are known to show biomass heterosis in their crosses [6], were used.

In our systems biological network hypothesis for heterosis [3], [4] partial correlations were used to characterize the global interaction structure of regulatory networks from observational time series data [7]. The hypothesis is based on the understanding of the heterosis phenomenon as increased adaptability as proposed in its basic idea by Shull [1] and, in particular, by Robertson and Reeve [8]. We expect a higher number of regulatory possibilities in the hybrids compared to the homozygous parents [8]. In our network hypothesis for heterosis, we extend the findings by Robertson and Reeve [8] in the way that we assume that more regulatory possibilities go along with more regulatory interactions on the molecular level in the heterozygous genotypes. According to our hypothesis, this increase in the connectivity of the regulatory networks can be detected as an increase in the significance of the partial correlations between the genes in either hybrid compared to the homozygotes. So, according to our network hypothesis for heterosis, we expect that heterozygous individuals, which are known to show heterosis, have denser regulatory networks which can be measured as increased significance of the partial correlations. For each of the two reciprocal hybrids we obtained a list of genes ranked according to the increase in significance of its partial correlation to each other gene compared to the mean of the parents (MPH) or the better of the two parents (BPH).

The top ranked genes of each of these ranking lists are compared to the list of genes from QTL experiments which identified genomic regions involved in biomass heterosis [5].

On the one hand, studies e.g. by Krieger *et al.* [9] suggest that *single* heterozygous mutations may improve productivity in agricultural organisms. In this case, only a few genes causally related to biomass heterosis are expected within each QTL region. Therefore, in our study, we would expect that the overlap between the genes identified in the QTL regions and the top ranked genes from the systems biological approach would *not* be significantly larger than by chance. On the other hand, from the systems biological point of view, it is predicted that probably many genes are involved in the complex trait of biomass heterosis. Monforte and Tanksley [10] concluded that their data agree with the hypothesis that interactions among different genetic loci, possibly closely linked, cause heterosis. If the two approaches towards finding genes responsible for biomass heterosis in the early development of *Arabidopsis thaliana* would show a significantly larger overlap than by chance, it suggests that each of the identified heterotic QTL regions contains more than only a few genes influencing biomass heterosis.

The main objective in this study was to test if those genes that are detected by the systems biological approach for biomass heterosis are enriched within the detected heterotic QTL regions. This is done by applying an over-representation analysis (ORA) based on the hypergeometric distribution in which the significance of the overlap between the resulting gene lists of either approach is calculated [11], [12].

To analyze the distribution of genes contributing to biomass heterosis over all five *Arabidopsis thaliana* chromosomes, we ran a chromosome-wise ORA. Furthermore, ORA were applied to identify pathways which contain significantly more of the genes of the resulting candidate group of genes from both approaches than expected by chance.

## Results

We performed an over-representation analysis (ORA) to analyze if two different approaches towards biomass heterosis in *Arabidopsis thaliana* point to similar genes which are probably responsible for this heterotic phenotype. A significant enrichment of the resulting genes from one analysis in the other would suggest that this assumption is true and, therefore, more genes influencing biomass heterosis are within the identified heterotic QTL regions than expected. Each of the analyses was performed for the two heterozygous genotypes C24×Col-0 and Col-0×C24 as well as regarding MPH and BPH.

**Figure 1:**
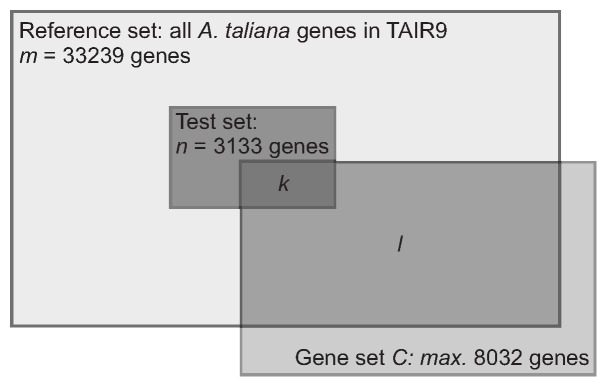
Setup of the over-representation analysis. It is test if a systems biological approach towards heterosis [4] and a quantitative genetics approach [5] point to similar genomic regions influencing biomass heterosis in the early development of *Arabidopsis thaliana*.

Our ORA (setup shown in Figure 1) was based on a reference set of all 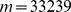 *Arabidopsis thaliana* genes in the TAIR database version 9 [13]. The test set was built out of the 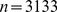 genes within the genomic regions that are involved in biomass heterosis determined in the quantitative genetics study by Meyer *et al.* [5]. Following Fury *et al.* [14], we used different numbers of genes in the gene set. Each gene set was created from the genes with the 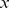 largest 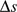-values (partial correlation heterosis effect values according to Eq. 2 and 3) separately for either hybrid as well as MPH and BPH. A large 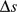-value indicates that the gene was identified in our systems biological analysis as probably involved in biomass heterosis in the early development of *Arabidopsis thaliana* [4]. For each number 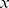 of genes (ranging from 0 to 8032 genes by steps of 100) we determined four gene sets 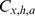 (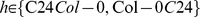 and 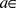 {MPH, BPH}).

The ORA was used to estimate the probability that the number of genes which overlap between the test set and the respective gene set is either due to chance or represents a true enrichment of the genes of the gene set in the test set.

**Figure 2:**
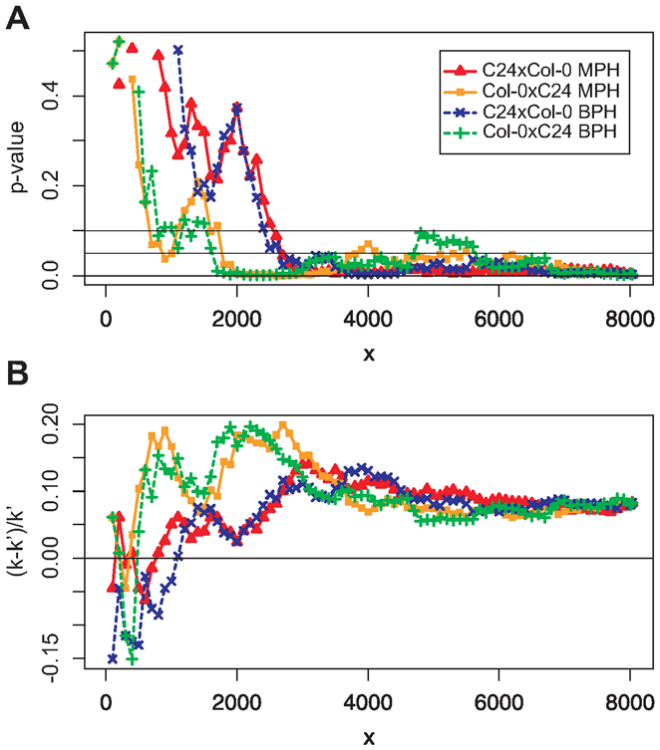
ORA results for gene lists of two different approaches towards biomass heterosis. 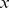
: number genes in gene set. (A) For gene sets of 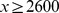 genes, both hybrids show a significantly larger overlap between test set (3133 genes determined in QTL mapping experiments for biomass heterosis [5]) and gene set (determined in systems biological network analysis [4]) than expected for a random test set for MPH as well as BPH. (B) Fraction of how much more genes are observed in the overlap between test set and each of the gene sets than expected if this overlap would be a chance event relative to the expected overlap. The over-representation is significant (A) but not very strong (B).

Figure 2A shows the results of the ORA. The x-axis depicts the number 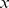 of genes which were used in the gene set of each particular ORA. The y-axis shows the probability (*p*-value according to Eq. 5) of having as many or more than the observed 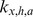 genes in the overlap between the 3133 genes in the test set and the 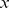 genes in the respective gene set if the genes of the test set would have been chosen randomly out of the reference set. For genotype C24×Col-0 for all gene sets 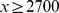 significantly (significance level 0.05) more genes were detected in both approaches than expected by chance for MPH as well as for BPH (Figure 2A). *P*-values were only calculated for the case where the observed number of genes in the overlap between test set and gene set was larger than we expected just by chance.

The result for the other hybrid, Col-0×C24, was similar. For 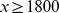 for MPH and 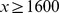 for BPH all ORA showed a significant overlap (significance level 0.1) between the results of the two different heterosis analyses. Different from C24×Col-0 not all gene sets with more than a certain number of genes resulted in a *p*-value 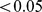. For MPH gene sets of 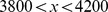 genes and for BPH gene sets of 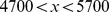 led to *p*-values between 0.05 and 0.1 (Figure 2A).

Figure 2B shows the fraction of how much more genes were observed in the overlap between test set and gene set (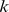 genes) than expected just by chance (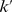 genes) in relation to 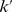. For large 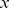-values we determined a constant percentage of more genes than expected by chance.

Summarizing, we identified a significant over-representation of the gene set in the test set (Figure 2A), but the enrichment was not very strong. A maximum of around 20% more genes in the overlap than expected just by chance was detected and an average of a little less than 10% for gene sets of 4000 genes or more (Figure 2B).

**Figure 3:**
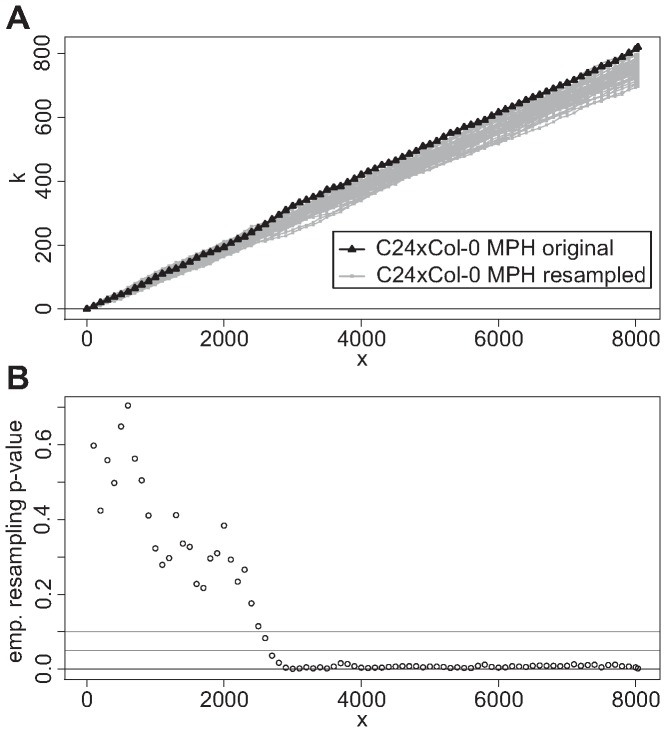
ORA with original and several random gene sets. This study was performed to confirm a significant over-representation between genes of two different approaches. (A) shows for C24×Col-0 MPH the number (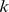) of genes in the overlap between 3133 genes in the test set and 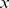 genes in the “original” gene sets (black) and in 50 randomly chosen gene sets (gray), respectively. For 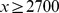 the calculated empirical resampling *p*-values on the basis of 1000 randomly chosen gene sets confirm a significant over-representation of the gene set in the test set (B).

As a second validation of the significance of the observed enrichment, we ran a resampling analysis in which the genes in the gene set were sampled 1000 times randomly out of all genes in the reference set. Figure 3A shows the relation between the number of genes observed in the overlap between test set and each “original” gene set and in the overlap to the resampled gene sets for MPH of genotype C24×Col-0. For the reason of clearness the results of only the 50 first resamplings were plotted. For 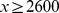 the number of genes in the respective “original” gene set also present in the test set exceeded the overlap between test set and nearly each randomly resampled gene set, confirming the findings from Figure 2A. This result is hardened by significant (significance level 0.05) corresponding empirical *p*-values, calculated for 1000 resamplings, for all 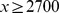 (Figure 3B).

The calculation of the empirical resampling *p*-values for BPH C24×Col-0 as well as MPH and BPH of Col-0×C24 also confirmed the result of a significant over-representation for sufficiently large 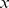 shown in Figure 2A.

To study if the determined significant over-representation for sufficiently large gene sets is the same over the five chromosomes in *Arabidopsis thaliana*, we ran the ORA separately for each chromosome. Again, the genes from within the regions that were determined during the QTL analysis by Meyer *et al.* [5] applying LOD-score thresholds for the empirical significance level of 5% were chosen as test set. As before, the gene sets were based on the results from the systems biological analysis by Andorf *et al.* [4]. However, in this analysis each gene set 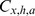 was split up into five gene sets (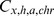). Each gene set contained only genes belonging to one of the five chromosomes.

**Figure 4:**
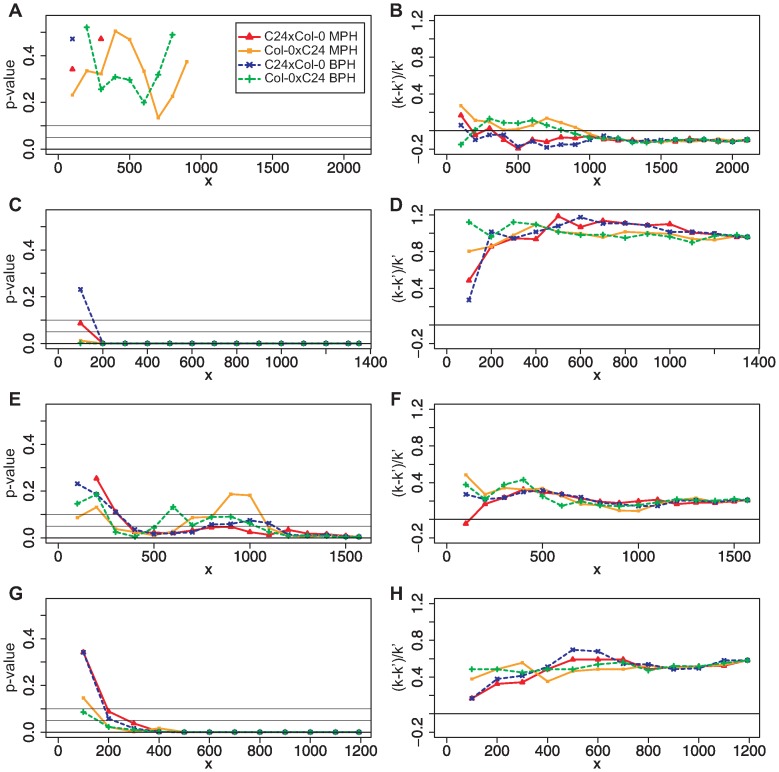
ORA for gene sets containing genes of only one of the five *Arabidopsis thaliana* chromosomes. 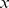
: number genes in gene set. (A)+(B): chromosome 1; (C)+(D): chromosome 2; (E)+(F): chromosome 3; (G)+(H): chromosome 4; for chromosome 5 no over-representation at all was observed and, therefore, no plots are shown. The probabilities of having as many or more genes in the overlap of random test sets to the gene set than observed for the experimental data are shown on the left side. The proportion of genes that were more in the overlap than expected by chance are shown on the right side. Genes on chromosomes 2 and 4 show a significant over-representation between the results of the two different approaches towards biomass heterosis in early development.

**Figure 5:**
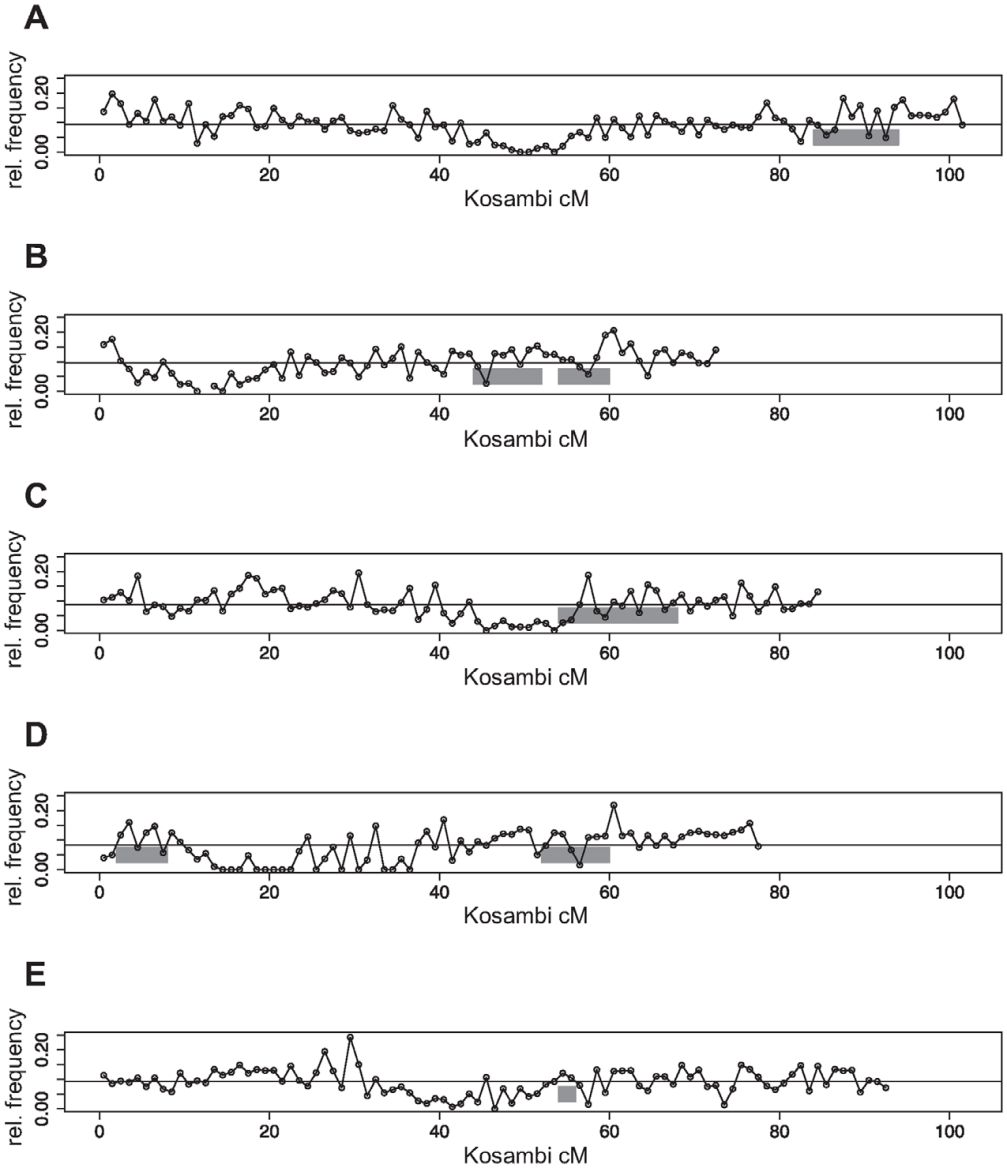
Relative frequencies of top ranked genes according to systems biological approach vs. Kosambi cM. Relative frequencies (fraction between the number of detected 3000 top ranked genes of C24×Col-0 MPH of the systems biological analysis and all known genes in *Arabidopsis thaliana* at a specific section on each chromosome) are plotted against the genetic distance (Kosambi cM). (A–E): chromosome 1–5. The gray boxes are the QTL candidate genomic regions contributing to biomass heterosis [5]. The horizontal lines are the median values of the relative frequencies per chromosome. For chromosomes 2 and 4 the relative frequency exceeds the median relative frequency especially in the area of the detected QTL. This is in line with the detected significant over-representation on these chromosomes of the genes detected in the systems biology approach in the genes from the QTL study.

The results of these chromosome-wise ORA are shown in Figure 4. For chromosomes number 1 (Figures 4A and 4B) and 5 no significant over-representation of the gene set in the test set was detected for either hybrid as well as heterosis measure, independent of the gene set size. For chromosome number 5 the observed overlap between the results of the two different approaches was for each number 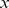 of genes in the gene set smaller than expected just by chance. Therefore, no plots for chromosome number 5 are presented in Figure 4.

Chromosomes 2 (Figures 4C and 4D) and 4 (Figures 4G and 4H) showed a significantly larger overlap than expected by chance between test set and gene set for both heterozygous genotypes and both heterosis measures for nearly each gene set size. For chromosome 3 the result was not as clear as for the other ones. The hybrid C24×Col-0 showed a significant enrichment (significance level 0.1) of the gene set in the test set for gene sets of 400 or more genes for MPH and BPH. The determined *p*-values for the ORA of the genotype Col-0×C24 fluctuated with different gene set sizes between significant on the level of 0.05, significant on the level of 0.1 and not significant at all. Hence, for chromosome number 3 only C24×Col-0 showed a significantly larger overlap between the results of the two approaches than expected by chance. However, this over-representation is much weaker than for chromosomes 2 and 4 (Figures 4D and 4H).

The plots in Figure 5 are based on the 3000 genes with the highest 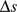-values for C24×Col-0 MPH. The x-axis shows the genetic distance (Kosambi centimorgan (cM)) of each of the five *Arabidopsis thaliana* chromosomes (5A–E: chromosomes 1–5). The determined heterotic QTL regions are represented as gray boxes. To show that for some chromosomal sections more of the 3000 genes were detected than expected by chance, we calculated relative frequencies as the number of the 3000 genes in a certain section of Kosambi cM, divided by the number of all known *Arabidopsis thaliana* genes from TAIR9 in this section. By building the relative frequencies we accounted for the different gene densities at diverse chromosome regions. These relative frequencies are shown on the y-axis. The horizontal lines are the median values of the relative frequencies for each chromosome. The detected overall over-representation was weak (Figure 2B) but particular significant on chromosomes 2 (Figure 4C) and 4 (Figure 4G). In the plots of these two chromosomes (Figures 5B and 5D) more points are above the respective median relative frequency values in the areas of the QTL than in other regions on the respective chromosome. Besides these sections that overlap with the QTLs, there are other enriched regions that where not detected in the QTL analysis.

Following Kliebenstein [15], we analyzed if the presence of candidate duplicated genes, which were found to belong more often to expression or phenotypic QTL than single copy genes, could be influencing the degree of overlap between the two different approaches towards biomass heterosis in our study. Using the information from Kliebenstein [15] about which genes are duplicated, we run additional over-representation analyses to determine the enrichment of these duplicate genes in the top 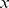 genes with the highest 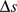-values from the systems biological analysis. Each hybrid, and MPH as well as BPH, show a significant enrichment (*p*-value 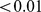) of the duplicated genes in each set of genes with 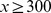. On the other side, the duplicated genes are not significantly enriched in the genes within the heterosis QTL regions.

Furthermore, it was analyzed to which functional group within the *Arabidopsis thaliana* plants the genes in the overlap between the two approaches towards biomass heterosis in *Arabidopsis thaliana* belong. This was done by using an ORA with a different setup than before. In this analysis, the overlapping genes between the 3000 genes with the highest 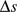-values, again separately for each hybrid and heterosis measure, and the 3133 genes identified in the quantitative genetics analysis were used as test sets. The reference set were all 8032 genes that were analyzed in the systems biological study according to Andorf *et al.* [4]. Each of the 80 gene sets that were used contained genes that belong to one pathway based on PO terms [16] or MapMan [17] which in turn is based on the TAIR8 database. Table 1 shows ranked lists of pathways with a significant over-representation (FDR corrected *p*-value 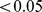). For the test set of Col-0×C24 BPH, no pathway with a significant enrichment was detected. The majority of pathways that showed a statistically significant over-representation are leaf or otherwise biomass related.

**Table 1:** Ranked results of ORA of overlapping genes between two approaches towards biomass heterosis and pathways of TAIR8 and PO.

C24×Col-0 MPH	C24×Col-0 BPH	Col-0×C24 MPH
LP.08 eight leaves visible	LP.08 eight leaves visible	petiole
LP.12 twelve leaves visible	leaf lamina base	LP.02 two leaves visible
petiole	LP.12 twelve leaves visible	leaf lamina base
leaf lamina base	petiole	LP.12 twelve leaves visible
shoot	shoot	F mature embryo stage
LP.02 two leaves visible	LP.02 two leaves visible	LP.08 eight leaves visible
cotyledon	cotyledon	
male gametophyte	F mature embryo stage	
F mature embryo stage		
guard cell		

The fundamental data (such as the 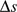-values) and results of the analyses are available upon request.

## Discussion

In this study two different approaches to determine genes that contribute to biomass heterosis in early development of *Arabidopsis thaliana* were integrated. We could show that the quantitative genetics approach by Meyer *et al.* [5] and the systems biological analysis by Andorf *et al.* [4] point to similar genomic regions influencing heterosis for biomass. An over-representation analysis (ORA) revealed that the resulting genes of these two studies showed a significantly larger overlap than expected by chance (Figure 2A). This suggests that not only single but several genes that are involved in biomass heterosis are located within the QTL regions that were detected in the quantitative genetics approach.

The result of a significant over-representation achieved in the parametric ORA was confirmed in a resampling analysis in which the genes of the gene set were randomly chosen 1000 times out of the reference set (Figure 3). However, while the enrichment was significant, it was not very strong (Figures 2B and 5). This result was achieved for either hybrid (C24×Col-0 and Col-0×C24) regarding both heterosis measures (MPH and BPH) (Figure 2) and markedly for two out of the five chromosomes (Figure 4).

In further ORA with a different setup we analyzed if the genes in the overlap between the results of the two approaches show a significant enrichment in one or more of 80 *Arabidopsis thaliana* pathways. The majority of pathways that showed a significantly larger overlap than by chance are leaf or otherwise biomass related (Table 1). This result is in line with an earlier analysis by Meyer *et al.* [6], in which they detected heterosis in the trait of biomass in early development of the same *Arabidopsis thaliana* accessions that were under study in this work.

Some methodological details of our approach remain to be discussed. The number of genes determined in the quantitative genetics approach was fixed due to the preselected criterion of an empirical significance level of 5% for the LOD-score thresholds. The number of genes in the results list of the systems biological approach was not specified for the ORA. The ORA was run several times to study the overlap between the 3133 genes of the QTL mapping experiments (test set) and the top ranked 0 to 8032 genes of the systems biological analysis (gene set).

We did not run the ORA for a fixed size of the gene set because Fury *et al.* [14] stated that the overlapping probability determined in ORA studies depends on the number of genes in the gene lists (test set and gene set) which are compared. Fury *et al.* [14] have shown that the overlapping significance increases (*p*-values decrease) with increasing number of genes in the gene set (or test set). So, small gene set sizes have a small overlapping significance. This may be one of the reasons why the *p*-values for low numbers 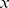 of genes in the gene set are not significant in our analysis (Figure 2A). In contrast, if no true signal (over-representation) is present, the gene list size does not effect the *p*-values that are observed [14].

If 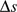-values for each gene in the reference set (all *Arabidopsis thaliana* genes in the TAIR9 database) of our ORA were available, the analysis could have been extended to 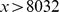 genes in the gene set. In this case we expect that the *p*-values of the over-representation would increase for larger gene set sizes 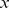 until it is not significant at all [14].

In the microarray experiments of the systems biological analysis 44k gene models were measured but reduced due to filtering steps and methodological reasons to only 8032 genes for this current work. The filtering involved a step in which genes that show no significant time and/or genotype-time point interaction effect in the applied linear model were excluded from the subsequent analysis [4]. This leads to the fact that in the 8032 genes in this study, the genes that are probably involved in biomass heterosis are already slightly enriched. This may be one reason for the significant over-representation for large 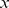-values shown in Figure 2A.

Furthermore, we want to point out that the results of the chromosome-wise analyses have to be used with care because each of the gene sets was relatively small. This chromosome-wise analysis can, therefore, not give a firm insight into which of the chromosomes contain the genes that are mainly responsible for biomass heterosis.

Over all, in our point of view, the use of the ORA is a legitimate approach to compare the two different datasets in this study. The genetic difference between the two homozygous parents with respect to specific locations in their genome is the basis for a possible heterosis effect. If this genetically different location is originating from duplicated genes or not, does not bias either the QTL or the systems biological approach.

In this work, an integrative analysis was presented. In comparison to the results of one experimental technique, the integration of two different experimental techniques accounts for the technological bias of each approach and the restriction to the particular level of biological information that is addressed by the single technique. The results that are found in an integrative analysis are more likely to be significant than the results of one single experimental technique as discussed in Steinfath *et al.* [18].

If only one to two genes per heterotic QTL region influence biomass heterosis in *Arabidopsis thaliana*, no significant over-representation between the results of the two approaches integrated in this work could be expected. However, we could determine a significantly larger overlap between the resulting candidate gene lists for biomass heterosis in the early development of *Arabidopsis thaliana* of the systems biological approach and the quantitative genetics analysis than expected by chance. This suggests that each heterotic QTL region contains several genes that influence biomass heterosis in *Arabidopsis thaliana*. The suggestion of several genes that influence heterosis within each heterotic QTL region that resulted of our ORA can be seen in line with the proposed model of pseudo-overdominance to explain the heterosis phenomenon. This model describes the situation of tightly linked genes with favorable dominant alleles linked in repulsion, i.e. dominant and recessive alleles on opposite homologues, leading to complementation and apparently overdominance in the hybrid [19]. Even though overdominance and pseudo-overdominance are very difficult to distinguish, several heterosis studies provided evidence that an effect of pseudo-overdominance exists [20].

Pseudo-overdominance is not the only cause of heterosis that is supported by our network hypothesis for heterosis. The hypothesis of epistasis as cause of heterosis, as shown in several studies (e.g. by [21]), is also in line with our findings.

Not each section on the chromosomes that seems to show an enrichment of candidate genes which were identified in the systems biological approach, was also detected in the quantitative genetics analysis (Figure 5). One reason might be that the density of genes that influence heterosis according to our network hypothesis for heterosis is too low at these regions to be detected by the quantitative genetics analysis. The other way, regions that show several genes identified in the systems biological approach, but which are not detected in the quantitative genetics analysis, could be “false positives” with regard to the systems biological approach. These may also play another role for heterosis, but arise as false positives in our context, as QTL analysis was performed specifically with regard to biomass. Hence, genes that are solely detected in the systems biology approach could be detected because they showed additional or increased correlations in their gene expression profiles as a result of other effects in the hybrids that are not related to biomass heterosis. These sections probably play an important role in the result of the weak over all enrichment between the detected genes in both approaches.

In an enrichment analysis of genes that are found to be duplicated [15], we showed that these duplicated genes are significantly enriched in the results of the systems biological approach but not in the genes within the QTL regions. Therefore, while these duplicated genes probably have some influence on the outcome of the ORA in this work, they are not the main, exclusive reason that led to the significant enrichment between these two approaches. If that would be the case, we would expect to observe a significant enrichment of the duplicated genes in either candidate gene sets (QTL approach and systems biological approach).

Furthermore, the genes within the overlap between the two experimental techniques are, in turn, significantly enriched in biomass related *Arabidopsis thaliana* pathways. This strengthens the assumption that more genes (not only the expected few genes) from within each QTL region are somehow involved in biomass heterosis. So, most probably several genes in the respective regions led to the detection of each heterotic QTL region. Furthermore, this significant enrichment is in line with the hypothesis that functionally related genes are rather adjacent on the chromosomes than randomly distributed. Riley *et al.* [22] proposed that the distribution of molecular functional classes of genes in *Arabidopsis thaliana* is not locationally independent. If functionally related genes influencing biomass heterosis would be distributed randomly over the chromosomes, no significant over-representation would have been detected in this work.

For biomass heterosis in *Arabidopsis thaliana* at 15 days after sowing the found QTLs account for only up to around 30% of the phenotypic variation [5]. This may be one reason, why the identified over-representation was weak even though it was significant. Perhaps, more genes influencing the phenotypic variation were identified using the systems biological approach but these genes were not detected in the QTL mapping experiments. An additional reason may lie in our systems biological network hypothesis for heterosis [4]. It is based on the idea that an increase in the significance of partial correlations indicates the existence of more regulatory interactions. However, we cannot rule out other reasons for the enlarged number of significant partial correlations in principle but this hypothesis was discussed in detail in Andorf *et al.* [4] and we adopt it as working hypothesis for the current manuscript. Another reason for this weak over-representation may be that *both* analyses that are integrated in this work identified several genes that do not affect the phenotypic variation. These genes which are not directly involved in biomass heterosis most probably differ in both approaches and, therefore, they do not overlap in the integrative analysis. The exclusion of these not overlapping genes is one aim of integrative analyses and may on the other hand be the reason for the weakness of the detected over-representation in this study.

Hence, the result of the integrative analysis not only points to several genes within the heterotic QTL regions influencing biomass heterosis but it also suggests that the identified overlapping genes can be seen, with an increased confidence compared to the results of only one experimental technique, as a candidate group of genes which are likely to be involved in the molecular basis of biomass heterosis of early development of *Arabidopsis thaliana*.

## Materials and Methods

### Genes According to a Systems Biological Analysis

To contribute to the understanding of heterosis, we proposed a systems biological hypothesis on the basis of molecular network structures [3], [4]. Our network hypothesis for heterosis is based on Robertson and Reeve [8], who suggested that heterozygous individuals carry a greater diversity of alleles and are therefore likely to contain additional regulatory possibilities compared to their homozygous parents. These additional regulatory possibilities lead to a higher adaptability of the hybrids and, thus, the heterosis phenomenon. Our hypothesis is further rested upon Werhli *et al.* [7], who suggested the use of partial correlations of features of time series profiles to estimate regulatory interactions. Following Robertson and Reeve [8] and Werhli *et al.* [7], we expect in our network hypothesis for heterosis that heterozygous genotypes which show heterosis contain more regulatory interactions and, therefore, denser regulatory networks than the homozygous parents. These additional regulatory interactions lead to an increase in the significance of partial correlations of features in the regulatory networks of the hybrids compared to the homozygous genotypes. A detailed discussion of our network hypothesis for heterosis can be found in Andorf *et al.* [4].

This network hypothesis for heterosis was tested and confirmed on transcriptome profiles from seven time points during the early development of two different homozygous *Arabidopsis thaliana* accessions (C24 and Col-0) and the two corresponding hybrids [4]. These heterozygous plants are known to show a heterosis effect in their biomass phenotype [6]. The microarray data that was used in this analysis is MIAME compliant and the raw data is available on http://www.ncbi.nlm.nih.gov/geo/under the accession number GSE30398.

For details about the experimental data and raw data preparation, see Andorf *et al.* [4]. Slightly different from the analysis described in [4], another ANOVA model was applied in this study along with a cutoff of 0.3 for the corrected *p*-values of the effects in the used linear model. This way only genes that show nearly no time dependency and/or genotype-time point interaction were excluded.

Different from the analysis described in Andorf *et al.* [4], in which several representative samples of 1000 genes each were analyzed, all genes remaining after filtering were processed at once in this work. Separately for each genotype, the partial correlations of the time profiles for each pair of these genes were calculated using the *R* package *GeneNet* [23], [24]. Along with the partial correlation values itself, two-sided *p*-values (null hypothesis: zero partial correlation) were calculated and FDR corrected according to Benjamini and Hochberg [25].

Following Werhli *et al.* [7], a significant partial correlation symbolizes a probably present regulatory interaction between the two belonging genes. In order to have a high value for two genes, between which most probably a regulatory interaction exists, we built 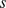-values:
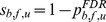
(1)

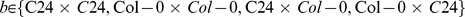
 denotes the genotype. 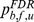 is the FDR corrected p-value of the partial correlation between the two genes 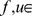 {1, …, 9263}.

Separately for each genotype for every gene the mean of its 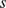-values to each other gene was computed. This way, we received one 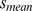-value for every gene for each of the four genotypes. On the basis of these 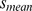-values we calculated the partial correlation MPH values 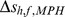 in respect of our network hypothesis as the difference between the 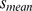-value of either hybrid to the mean of the 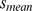-values of the homozygous genotypes:
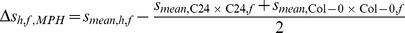
(2)where 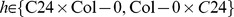 denotes the hybrid and 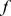 the gene.

Partial correlation BPH values (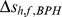) were calculated as the difference between the 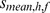-value and the larger of the 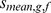-values of the two homozygous genotypes (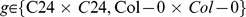) for each gene 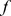:
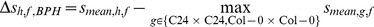
(3)


A high 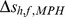-value is achieved when the gene is probably involved in more interactions in the regulatory network of the respective hybrid than it is expected in the mean of the two homozygous parents. Correspondingly, a high 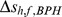 suggests that the gene is involved in more regulatory interactions in the hybrid than in the better of the two parents. Following our network hypothesis for heterosis, genes with high 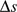-values are likely to be involved in biomass heterosis.

With the cutoffs used in this analysis, our hypothesis about additional regulatory interactions holds true for either hybrid regarding MPH as well as BPH. However, we want to state that this is not a direct candidate gene approach in the way that we expect that for example the 100 genes with the highest 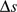-values are all involved in biomass heterosis. Instead of that, we can just establish that a list of genes with high 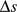-values contains various genes that may have an impact on biomass heterosis.

In our over-representation analysis (ORA) only genes which are identified with an AGI code can be used. Therefore, genes with unknown AGI code were excluded from the enrichment analysis. 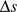-values of different gene models of one gene according to the TAIR9 database were averaged. This left 8032 genes with a known AGI code and a 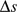-value for each hybrid and heterosis measure for the ORA.

### Genes According to a QTL Analysis

The genomic regions involved in early stage biomass heterosis were identified as described in Meyer *et al.* [5], using composite interval mapping (CIM) as implemented in the QTL mapping software PLABQTL [26]. Data were obtained from recombinant inbred line (RIL) populations [27] derived from the same *Arabidopsis thaliana* accessions C24 and Col-0 as used in the systems biological analysis described above. For the analysis, 838 testcrosses between the homozygous parents and 429 RILs were used.

Genomic regions were identified as having an influence on biomass heterosis, if the corresponding LOD-score exceeded the LOD threshold with an empirical significance level of 5%. LOD thresholds were determined separately for each trait by 5000 permutations [28]. The genes within the genomic regions were identified on the basis of the TAIR9 genome release (The Arabidopsis Information Resource, ftp://ftp.arabidopsis.org/home/tair/Genes/TAIR9_genome_release, June 2010) [13]. 3133 genes in 7 genomic regions constituted the test set for our ORA.

### Over-representation Analysis

In an over-representation analysis (ORA), it is studied if a list of genes (gene set) is over-represented (represented more than expected by chance) or under-represented (represented less than expected by chance) with respect to another gene list (test set). Furthermore, the probability is estimated how likely this over-representation or under-representation is due to chance considering a specific reference set of genes [11], [12]. In this work the experimental data is tested for over-representation only.

We used an ORA to test if two different approaches towards more insight into biomass heterosis in *Arabidopsis thaliana* point to similar genes. One approach was based on quantitative genetics [5] and the other on systems biology [4]. Each of these two studies led to a list of genes probably involved in biomass heterosis. While the QTL approach provided directly a list with 3133 genes due to the given LOD-score thresholds, the number of genes in the top ranked list based on the systems biological analysis’ was not fixed by a preselected criterion.

The setup of our ORA is shown in Figure 1. It is identical to an one-tailed version of Fisher’s exact test. The reference set consisted of all 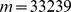 *Arabidopsis thaliana* genes listed in the TAIR database version 9. During the QTL study 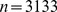 of these genes were identified as genes which are probably involved in biomass heterosis and we refer to these genes as the test set. A certain number (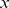) of genes detected in our systems biological approach to heterosis with the highest 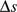-values (Eq. 2 and 3) built the gene sets 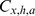 (separately for each hybrid 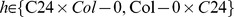 and heterosis measure 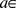 {MPH, BPH}). This led to four different gene sets for each 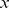. The gene sets contained a maximum of 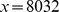 genes in case all genes, even with negative 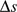-values, were used. The number of genes in the reference set which belong to gene set 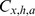 is denoted by 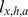. The number of genes of our test set which overlap with the current 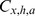 is given by 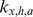.

Given 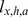, 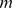 and 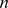, the number of genes (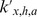) that would be in the overlap between test set and gene set 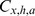 in the case that the test set is chosen randomly out of the reference set can be calculated:
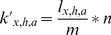
(4)


The genes of the gene set 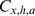 are called to be enriched in the test set if 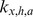 (genes observed in the overlap) is significantly larger than 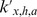 (genes expected in the overlap just by chance) [12].

A hypergeometric distribution was used to estimate the probability of observing an overlap of 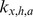 genes between test set and gene set if these sets were independent (null hypothesis) [11], [14]. The probability (one-sided *p*-value) of having as many or more than 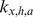 genes in the overlap between test set and gene set 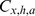 can be calculated by summing up the probabilities of having 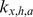 or more genes belonging to the test set also in the gene set in the case that the genes of the test set are randomly chosen from the reference set [11]:
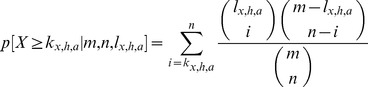
(5)where 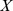 is a hypergeometric distributed random variable giving the size of the overlap. A *p*-value smaller than a given significance level corresponds to a significant over-representation of the gene set in the test set.

This whole analysis was performed in *R* [29]. The function *phyper* was used to calculate the *p*-values corresponding to the hypergeometric distribution.

Fury *et al.* [14] stated that the overlapping probability in an ORA changes with the number of genes in the test set and gene set. In our study the number of genes in the test set was fixed to 3133, but the number of genes in the gene set was not fixed. Therefore, the ORA was run with different numbers of genes in the gene set to analyze the influence of the gene set size on the overlapping probability. For each run the genes with the 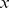 largest 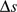-values were selected, separately for each hybrid as well as MPH and BPH, as the gene set. The number of genes in the gene set was ranged for the different ORA from 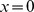 to 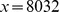 (all genes in the systems biological network analysis) by steps of 100.

### Resampling Analysis of Enrichment

In the ORA using a hypergeometric distribution, the probability to detect as many or more than the observed 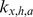 genes in the overlap between test set and gene set 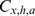 when a random test set is used is estimated. In a resampling analysis we calculated empirical resampling *p*-values on the basis of a resampling of the genes in the gene set. For each number 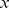 of genes in the gene sets we sampled new genes out of all genes in the reference set without replacement and assigned them to the original 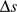-values. This is done 1000 times for any 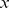. For each of these random gene sets the number of genes of the test set also present in the gene set were determined (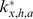). The number of genes in the overlap between each original gene set and the test set is depicted by 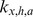. We used these values to calculate for each 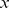 the empirical resampling *p*-values:
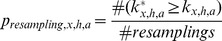
(6)


Empirical resampling *p*-values (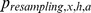) smaller than a given significance level are achieved in the case where the original gene set leads to a significantly larger overlap to the test set than expected by chance. This approach does not require distributional assumptions.

### Chromosome-wise Over-representation Analysis

We analyzed if genes which are involved in biomass heterosis are functionally located at only some of the five *Arabidopsis thaliana* chromosomes.

In this study for any number 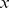 of genes in the “original” gene set, five ORA were performed. Each time the gene set 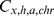 contained only the genes detected in our systems biological approach towards heterosis which belong to one of the five chromosomes (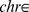 {1, …, 5}). The assignment to the chromosomes was done based on the TAIR9 database. The reference set and test set of these ORA were the same as before.

Of all 8032 genes that were analyzed in the systems biological approach, 2105 belong to chromosome number 1, 1348 to chromosome 2, 1569 to chromosome 3, 1193 to chromosome 4 and 1807 to chromosome 5. 10 of the 8032 genes under study are not listed in the TAIR9 database.

### Pathway Analysis of Candidate Group of Genes

In an attempt to get a little further insight into the molecular basis of biomass heterosis we determined the functional assignments of the genes in the overlap between the resulting genes from the systems biological analysis according to Andorf *et al.* [4] and the genes determined in the quantitative genetics approach by Meyer *et al.* [5]. This functional enrichment analysis was done by applying four further ORA. This time the reference set were all 8032 genes analyzed in our systems biological analysis. Four different test sets were used; one for each hybrid-heterosis measure combination. Each test set was built by first determining the 3000 genes with the highest 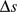-values. Then the overlap of these 3000 genes to the 3133 genes detected in the quantitative genetics approach was identified and used as test set.

As gene sets we used 80 *Arabidopsis thaliana* pathways which contain between 10 and 4000 of the 8032 genes in the reference set. 28 of them were based on MapMan [17], which in turn is based on the TAIR8 database. The remaining 52 pathways were built using Plant Ontology (PO) terms [16].

The *p*-values achieved in this ORA were corrected for multiple testing using the FDR approach by Benjamini and Hochberg [25].

In this setup we could determine if the overlapping genes between the two analyses are significantly enriched in one or more pathways (functional groups) of *Arabidopsis thaliana*.
